# Student Employability in the Transition from University to the Labor Market: The Role of Faculty Support and Self-Compassion

**DOI:** 10.3390/ijerph23050557

**Published:** 2026-04-25

**Authors:** Giovanni Schettino, Maria Francesca Trocino, Ilaria Poderico, Vincenza Capone

**Affiliations:** Department of Humanities, University of Naples “Federico II”, 80133 Naples, Italy

**Keywords:** employability, self-compassion, faculty support, career self-efficacy, career planning, well-being

## Abstract

**Highlights:**

**Public health relevance—How does this work relate to a public health issue?**

- The transition from university to the labor market is a critical period for students, marked by heightened employment uncertainty and significant perceived stress. From a public health perspective, this condition constitutes an area of psychosocial vulnerability that requires preventive strategies. Promoting resources such as self-compassion and fostering more supportive academic environments may strengthen students’ adaptive capacities, enhance their perceived employability, and mitigate the impact of career-related uncertainty during the transition to the labor market.

**Public health significance—Why is this work of significance to public health?**

- Identifying protective psychosocial resources among university students contributes to a preventive public health approach aimed at supporting adjustment during the university-to-work transition. By clarifying the mechanisms through which faculty support and self-compassion foster perceived employability, this study provides evidence on individual and contextual factors that can be targeted in higher education settings to strengthen students’ adaptive capacities in employment-uncertainty contexts.

**Public health implications—What are the key implications or messages for practitioners, policymakers and/or researchers in public health?**

- Higher education institutions and public health stakeholders should consider student employability as a psychosocial resource that can be strengthened through preventive interventions. University-based initiatives aimed at enhancing self-compassion, career self-efficacy, and autonomy-oriented teaching practices may support students’ adaptive functioning during the university-to-work transition.

**Abstract:**

In the current labor market, perceived employability is a key resource for university students approaching the transition from university to work, which is often marked by heightened stress, vulnerability, and unhealthy behaviors, particularly in contexts with high youth unemployment rates. Despite prior research documenting the buffering role of perceived employability in the relationships between career-related stressors and well-being, limited evidence exists regarding the roles of faculty support and self-compassion, a fundamental factor for effective emotional regulation, during university years. Consequently, this study aimed to examine the relationships between faculty support, self-compassion, career self-efficacy, career planning, and perceived employability through a self-report questionnaire completed by 186 Italian university students, mainly female, with a mean age of 21.24 (*SD* = 2.57). Results from a partial least squares model indicated that faculty support was indirectly associated with perceived employability through self-compassion, career self-efficacy, and career planning. These findings could support higher education organizations by suggesting the design of interventions to promote supportive learning environments and to develop training in emotional regulation skills. Such an approach could empower students to effectively cope with career-related stressors and, in turn, engage in adaptive behaviors associated with employability.

## 1. Introduction

Facing the labor market after completing university studies is a stressful experience for students, frequently characterized by high levels of anxiety and uncertainty [1,2]. Specifically, this crucial developmental stage requires students to address new demands despite lacking the necessary skills to manage them [3]. The above-mentioned issues are particularly critical in Italy, where graduate employment rates remain persistently below the European average [4]. This protracted school-to-work transition can be attributed to structural factors, including cyclical economic downturns and a pervasive mismatch between the competencies employers require and those embedded in university curricula [5]. The implementation gap is particularly acute concerning soft skills, which Italian academic programs often under-prioritize relative to disciplinary knowledge [6]. Therefore, this condition leaves Italian graduates particularly ill-equipped to face the challenges of protean careers [7]. Indeed, because this career approach relies on individual agency and the ability to cope with continuous role reconfigurations, universities’ lack of investment in improving soft skills may hinder students from effectively self-managing their professional trajectories. In this context of precariousness, students tend to report greater intolerance of uncertainty [8,9], a condition that can hinder career decision-making processes [10,11,12]. Following this line of reasoning, it is essential to identify the individual resources that enable effective coping with stressors such as those related to job-market insecurity [13]. Two key resources stand out: self-perceived employability [14] and self-compassion [15,16]. More specifically, both are fundamental for adapting to labor market instabilities, fostering better psychological adjustment, and promoting increased well-being [17,18]. These resources may also be fostered by instructors during the university years, as their support can enhance students’ autonomy, confidence, and proactive career behaviors [19,20]. However, the existing literature has largely underestimated the role of autonomy support as perceived by undergraduate students and its relationships with career success.

Therefore, the present study investigated them primarily through the lens of Social Cognitive Career Theory (SCCT) [21] in order to provide insights for developing targeted interventions that not only enhance students’ academic performance but also strengthen the personal resources necessary for the complex transition to the workforce.

### 1.1. Theoretical Framework

The SCCT [21,22] offers a comprehensive theoretical framework for understanding the mechanisms through which psychosocial resources related to career development translate into students’ adaptation to the labor market or their employability. It is rooted in Bandura’s [23] social cognitive theory, which conceptualizes individuals as active agents in shaping their careers and emphasizes the reciprocal interplay among personal, behavioral, and environmental factors.

Consequently, engagement in adaptive career behavior is shaped by key cognitive variables, including efficacy beliefs. In this regard, career self-efficacy refers to individuals’ beliefs about their ability to organize and perform the actions required to successfully address specific career-related tasks [24,25]. Such a belief acts as a motivational factor, as individuals with higher confidence in their competencies are more likely to anticipate positive outcomes and persist in the face of obstacles [26,27]. The literature has acknowledged the central role of career self-efficacy in career adaptation processes, showing its direct and indirect effects on young adults’ perceptions of employability [28]. Indeed, this factor strengthens confidence in one’s competencies and increases readiness to engage in complex and challenging career tasks [29]. In this regard, career self-efficacy can be considered a key psychological resource for enhancing employability [30] and has been positively associated with career decision-making self-efficacy and employability among university students [31].

Moreover, career self-efficacy has been acknowledged as a key variable associated with individuals’ confidence in managing career-related tasks and with greater engagement in goal-directed processes such as career planning [32], defined as the process of setting clear career goals and formulating strategies to achieve them [33,34]. For university students approaching graduation, planning is not merely a bureaucratic task but reflects proactive behaviors through which they organize their career choices and plan actions to construct their future occupational pathways, in relation to labor market opportunities and demands [35]. Notably, the period immediately preceding graduation is a critical phase of preparation for the university-to-work transition characterized by increased decision-making demands, a high influx of career-related information, and the need to accomplish key developmental tasks related to the construction of one’s professional future [34,35]. During this phase, defining clear career goals and developing strategies to achieve them signals the completion of an important developmental task [36] and constitutes a mastery experience that can strengthen personal efficacy beliefs in managing career-related challenges [23,37]. In this context, career planning has been linked to reduced career-related worry and enhanced career-related self-efficacy [34]. Additional evidence has indicated that career planning is positively associated with effort regulation in educational pathways and, when combined with autonomous motivational resources, contributes to improved academic outcomes [38].

This resource-based perspective aligns with the COR theory [39,40], which explains how individuals seek to build, maintain, and protect resources to cope with environmental demands. A core principle of this theory is that resources operate in dynamic cycles: gain cycles, in which existing resources promote the acquisition of new resources, and loss cycles, in which resource deficits increase vulnerability to further depletion. From this perspective, both contextual and personal resources contribute to activating developmental chains that support individuals’ adaptation in uncertain contexts, such as the transition from university to the labor market [34,35].

Based on this integrated framework, the present study proposes that faculty support serves as a contextual resource that promotes the development of personal resources, such as self-compassion and autonomy, which are associated with career self-efficacy. In turn, career self-efficacy fosters goal-directed processes, such as career planning and perceived employability [32,34]. The latter can, therefore, be understood both as the outcome of a process of resource accumulation [40] and as the result of socio-cognitive mechanisms [21,22], through which personal factors, cognitive variables, and contextual conditions interact to support students’ adaptation during the transition to the labor market.

### 1.2. Employability

To evaluate effective career management, employability stands among the key indicators in the literature [17,41]. It refers to individuals’ ability to obtain and maintain employment and to manage career transitions autonomously in response to labor market demands [42,43,44]. Consequently, employability transcends the mere potential to find a job, as it reflects individual agency in shaping career paths, thereby contributing to overall quality of life [45]. In other words, it can be understood as a dynamic set of resources accumulated through educational and professional experiences that remain relevant across career stages, particularly during periods of uncertainty [46,47].

Considering the subjective dimension of these assets, perceived employability has been framed as “the individual’s perception of his or her possibilities of obtaining and maintaining employment” [13] (p. 594). Therefore, perceived employability results from the interaction between individual factors, such as skills, abilities, and personal characteristics, and structural factors in the economic context and labor market [48].

This subjective assessment is particularly salient for university students, who must clarify career goals and evaluate the alignment between their competencies and labor market demands [42]. In the high-stress context of the university-to-work transition, the Gain Paradox Principle becomes particularly relevant: it is in situations of resource threat or uncertainty that acquiring new resources becomes vital to prevent a loss spiral, as the COR theory [49] states. Thus, perceived employability acts as a manifestation of a gain cycle, reflecting the successful mobilization of resources that support confidence and planning [50].

Existing literature identifies several antecedents of self-perceived employability. Among university students, core self-evaluations and job-search preparedness are positively associated with employability perceptions [51]. Similarly, individual and organizational factors—including career ambition, perceived university reputation, and academic commitment—foster these perceptions [17]. Furthermore, psychological resources such as efficacy beliefs and confidence in one’s competencies serve as further antecedents [52]. Finally, engaging in proactive career planning is consistently associated with a greater perceived capacity to enter the labor market [32,34].

Regarding the outcomes of self-perceived employability, evidence consistently shows that higher levels of self-perceived employability are associated with more favorable career outcomes, including greater job satisfaction and career success, as well as better psychological well-being [17,53,54,55,56].

### 1.3. Self-Compassion as a Psychosocial Resource for Career Development

Understanding perceived employability requires considering the combination of contextual and personal resources that could contribute to its development in university students. In this vein, among the personal resources that support self-regulatory mechanisms, self-compassion plays a distinctive role. It is defined as an adaptive way of relating to oneself that promotes kindness, nonjudgmental acceptance, and a balanced awareness of one’s emotional experiences [57,58]. It is a manifestation of a psychological process that supports the regulation of negative emotions and prevents the activation of dysfunctional responses such as harsh self-criticism, isolation, and rumination [18,59,60] by promoting adaptive coping styles [61].

Consistent with this thesis, Gilbert et al. [62] define self-compassion as “sensitivity to suffering in self […] with a commitment to try to alleviate and prevent it” (p. 1). In other words, it can be conceptualized as a motivational process oriented toward the regulation of suffering, based on individuals’ ability to recognize and tolerate their own pain and to implement responses that mitigate it.

In line with the COR theory, it is worth noting that resources do not operate in isolation but tend to cluster into resource caravans, in which the availability of specific resources fosters the acquisition and strengthening of others [63]. Notably, in the university-to-work transition, where students frequently encounter setbacks, such as rejected job applications or perceived skill gaps, self-compassion may be a factor that promotes the resource-preservation mechanism. Specifically, self-compassionate students, by approaching their anxiety or disappointment with a motivation to alleviate it, prevent the rapid depletion of psychological energy caused by harsh self-criticism or suppression. Consequently, this preservation of emotional resources allows them to reinvest their energy in adaptive problem-solving behaviors—such as seeking feedback, refining career plans, or upskilling—thereby converting a potential resource-loss spiral into a resource-gain cycle that supports sustained employability.

Additionally, by mitigating self-critical judgments, self-compassion enables students to reframe career setbacks as manageable learning experiences rather than reflections of personal inadequacy. In academic contexts, this perceived efficacy promotes adaptive self-regulation and fosters psychological well-being [59]. Specifically, highly self-compassionate students view failures as less threatening, which allows them to sustain their motivation and confidence [18]. Furthermore, self-compassion is consistently linked to lower anxiety, stress, and depression; decreased rumination; and higher life satisfaction [18,59,60]. Finally, regulating negative emotions through a self-compassionate attitude can enhance psychological adjustment and overall well-being during periods of career uncertainty [18].

Given the above, we expected positive associations between self-compassion, career self-efficacy, career planning, and perceived employability.

### 1.4. Faculty Support as a Contextual Resource

Given that perceived employability is fundamentally the result of self-directedness and individual agency [64], providing an academic environment that promotes students’ autonomy represents a critical prerequisite for their development. In line with the SCCT framework [37], which identifies contextual supports as critical facilitators that can directly encourage adaptive behaviors or, indirectly, strengthen them by increasing self-efficacy [65], the university context offers multiple forms of social support associated with students’ career development. Literature indicates that students’ academic adjustment, psychological well-being, and retention are affected by the quality of their social relationships at multiple levels, including peer networks and the broader academic community [66,67]. For example, Wilcox et al. [68] found that students’ successful transition and persistence are more strongly affected by the quality of their social interactions and friendships than by institutional structures alone. Similarly, Zarbo et al. [69] suggest that a strong sense of community significantly enhances academic satisfaction and well-being while acting as a critical protective factor against the risk of university dropout.

While these diverse support sources provide an essential foundation for individuals’ well-being [70], the teaching practices adopted by instructors to support students are an essential dimension of faculty support that have often predicted students’ academic performance and engagement [71]. Specifically, such support serves as a primary contextual resource that helps buffer students against stress and promote the acquisition of personal resources necessary for the transition to the labor market [20,72]. This factor can manifest mainly in instrumental aid (e.g., direct career advice) or in informational guidance [73]. However, when aiming to foster students’ proactive career construction, autonomy support is particularly salient [19]. Rooted in Self-Determination Theory (SDT) [74], this construct does not merely refer to a supportive presence. Rather, it consists of a specific interpersonal style in which the instructors acknowledge the students’ perspectives, legitimize their feelings, provide opportunities for choice, and minimize controlling practices [75]. In this sense, autonomy support constitutes a contextual resource that may foster student career development through specific psychological mechanisms.

According to the SDT [76], support for autonomy acts as an environmental resource that meets a basic psychological need (i.e., autonomy), thereby enhancing self-determination and, consequently, effectiveness in making career decisions [19]. Moreover, by conveying confidence in students’ abilities, instructors provide external validation that helps students to be aware of failures and difficulties without denying them, thereby improving the effectiveness of their coping strategies for facing negative emotions. This assumption aligns with the premise that receiving compassion is internalized by the individual, subsequently transforming into self-directed kindness [62]. Indeed, empirical evidence demonstrates that experiencing compassion from others [77] and high levels of perceived social support [28,78] are positively associated with self-compassion and inversely related to self-criticism [79].

This supportive climate may, in turn, encourage the development of self-efficacy beliefs. Notably, when instructors express trust in students’ potential, students’ confidence in their ability to manage complex career-related tasks increases [80,81]. In line with Gilbert’s theorization [82], experiencing such support may trigger the individual’s soothing affiliative system [83], which helps to downregulate threat-based reactions and promotes a more balanced self-evaluation. This process may lead to a progressive accumulation of emotional and cognitive assets [40], which can provide the resources students need to engage in proactive career planning [37]. In other words, by reducing the anxiety associated with external evaluative pressure, autonomy support can free up cognitive resources, enabling students to translate their agency into concrete actions, such as setting career goals and developing strategies to achieve them.

Therefore, we postulate that faculty support may be positively associated with self-compassion. The latter may be positively associated with career self-efficacy, which, in turn, may empower students to engage in proactive planning necessary for a successful transition into the labor market.

### 1.5. The Current Study

Given the considerations outlined above, the present study aims to investigate psychosocial mechanisms that foster the development of perceived employability among university students facing the complex transition to the labor market. Despite prior research widely documenting the role of organizational and personal resources in promoting career success, limited attention has been paid to how faculty support and self-compassion can trigger a resource-gain cycle [53] that leads students to implement adaptive career behaviors. Therefore, integrating the COR theory [49] and Lent and Brown’s [37] theorization, we postulated that autonomy-supportive behaviors from instructors may enhance students’ perceived employability by sequentially promoting self-compassion, career self-efficacy, and career planning.

**Figure 1:**
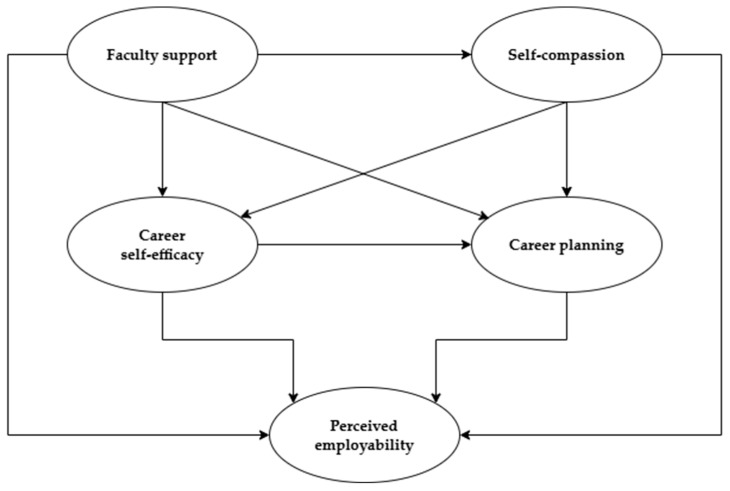
Hypothesized model.

Specifically, we proposed the model depicted in Figure 1 and postulated the following hypotheses:

**Hypothesis** **1** **(H1).** *Faculty support is positively associated with self-compassion (H1a), career self-efficacy (H1b), career planning (H1c), and perceived employability (H1d).*

**Hypothesis** **2** **(H2).** *Self-compassion is positively associated with career self-efficacy (H2a), career planning (H2b), and perceived employability (H2c).*

**Hypothesis** **3** **(H3).** *Career self-efficacy is positively associated with career planning (H3a) and perceived employability (H3b).*

**Hypothesis** **4** **(H4).** *Career planning is positively associated with perceived employability.*

**Hypothesis** **5** **(H5).** *The relationship between faculty support and perceived employability is serially mediated by self-compassion, career self-efficacy, and career planning.*

## 2. Materials and Methods

### 2.1. Participants and Procedure

This study adopted a cross-sectional survey design. Participants were recruited using a non-probability convenience sampling approach and completed an online self-report questionnaire via the Qualtrics platform. The survey took participants approximately 15–20 min to complete. All items were set to mandatory to prevent missing data and ensure complete responses from all participants. Data were collected between October and December 2024. Participation in the study was voluntary, and the anonymity of subjects was guaranteed in accordance with the General Data Protection Regulation and the Helsinki Declaration [83]. In order to complete the questionnaire, individuals provided informed consent and were assured that their data would be processed exclusively for research purposes. Responses were combined in an aggregated manner without any possibility of identifying the personal information of subjects. No incentives were provided for completing the questionnaire.

Participants were invited through the online academic platforms used for the lessons. Moreover, to minimize social desirability bias, the questionnaire was completed by the students outside of regular class hours. Neither instructors nor researchers were present during the administration process, ensuring a pressure-free environment, and respondents could withdraw from the study at any time without consequences. The adequacy of the sample size was assessed using a priori power analysis in accordance with established guidelines for PLS-SEM [84]. The sample size needed to detect a minimum path coefficient of at least 0.20 with a statistical power of 80% was computed. More precisely, the inverse-square-root method indicates a minimum sample of 155 participants. Among the invited participants, a total of 186 Italian university students enrolled in psychology degree programs met the inclusion criteria and completed the questionnaire. Thus, our sample provided adequate statistical power to evaluate the structural model and the hypothesized paths.

Regarding demographics, 67.2% of participants were female, aged 18–33 years (*M* = 21.24; *SD* = 2.57). The majority of participants (74.7%) were second-year students enrolled in a three-year bachelor’s degree program. Regarding educational background, 81.2% of participants held a high school diploma as their highest qualification, while 18.8% had previously completed a bachelor’s or master’s degree in another field before enrolling in psychology. Concerning occupational status, 78.5% were full-time students or job seekers, while 21.5% were employed (either as employees or self-employed). Additionally, 67.7% of participants reported an average of 3.1 work experiences (*SD* = 2.4). Regarding socioeconomic status, 75.8% reported a middle- to high-SES level. Finally, participants reported moderate levels of self-perceived academic success (*M* = 6.88; *SD* = 1.99).

### 2.2. Measures

Whenever available, validated Italian versions of the scales were utilized to ensure cross-cultural reliability. When these validations were not available, the items were independently translated by three bilingual researchers and then jointly reviewed to reach consensus. Therefore, after providing informed consent, participants completed the following measures in a fixed order.

*Faculty support* was measured using the Learning Climate Questionnaire (LCQ) short version [76], validated in Italian by Monacis et al. [85]. The LCQ is designed to assess students’ perceptions of autonomy support from their instructors. The short form contains six items measured on a 7-point Likert scale ranging from 1 = “*strongly disagree*” to 7 = “*strongly agree*” (e.g., “*My instructors conveyed confidence in my ability to do well in the course*”). Cronbach’s *α* = 0.89.

*Self-compassion* was assessed using the Self-Compassion dimension of the Italian validation (SC-CEAS) [86] of the Engagement and Action Scales (CEAS) [62]. The scale consists of 13 items assessing self-compassion. A higher score indicates a greater degree of self-compassion. Participants rated each item on a scale ranging from 1 = “*never*” to 10 = “*always*” (e.g., “*I take the actions and do the things that will be helpful to me*”). Cronbach’s *α* = 0.88.

*Career self-efficacy* was measured using the Career Self-Efficacy Scale [87]. The scale consists of 7 items assessing perceived confidence in one’s ability to perform career-related tasks. A higher score indicates a greater degree of career self-efficacy. Participants indicated their degree of agreement on a 5-point Likert scale ranging from 1 = “*strongly disagree*” to 5 = “*strongly agree*” (e.g., “*I believe that I can do what I need to do in order to make my career successful*”). Cronbach’s *α* = 0.81.

*Career planning* was assessed using the 6-item Career Planning Scale by Gould [33], which measures the degree of an individual’s career planning. Specifically, the scale evaluates the extent to which a person has formulated clear, specific career plans and has strategies to achieve them. A higher score indicates a greater degree of career planning. Participants rated each item using a 5-point Likert scale ranging from 0 = “*strongly disagree*” to 4 = “*strongly agree*” (e.g., “*I have a well-defined strategy for achieving my career goals*”). Cronbach’s *α* = 0.79.

*Perceived employability* was evaluated using five items from Berntson and Marklund [88]. The scale assesses individuals’ perceptions of their possibilities of obtaining employment. A higher score indicates a greater degree of perceived employability. Participants rated each item on a 5-point Likert scale ranging from 1 = “*not at all*” to 5 = “*completely*” (e.g., “*My personal qualities would make it easy for me to get a job*”). Cronbach’s *α* = 0.79.

The questionnaire included a sociodemographic section in which participants were asked to provide their age, gender, year of enrollment, previous educational qualifications, region of residence, socioeconomic status, current employment status, work experience, and academic achievement.

### 2.3. Statistical Analyses

To evaluate the hypothesized serial mediation model, we adopted a Reflective Partial Least Squares Structural Equation Modeling (PLS-SEM). This technique was chosen for its established effectiveness in estimating complex path models with non-normal data and relatively small sample sizes, as documented by Hair et al. [89]. The PLS-SEM analysis followed a two-step procedure: the assessment of the measurement (outer) model and the evaluation of the structural (inner) model. Control variables (age, gender, work experience, socioeconomic status, and academic success) were included in the structural model as direct predictors of employability to account for potential confounding effects. Significance levels for path coefficients were determined through a bootstrapping procedure with 10,000 subsamples, using percentile bootstrap confidence intervals with a fixed seed to ensure reproducibility and stability in parameter estimation. We evaluated indicator reliability by examining factor loadings; items with loadings below 0.50 were considered for removal. Internal consistency reliability was assessed using Cronbach’s alpha (α), Dijkstra–Henseler’s Rho_A, and composite reliability CR (all thresholds > 0.70) [90]. Convergent validity was verified by examining the Average Variance Extracted (AVE), which should exceed 0.50 [90]. Discriminant validity was assessed using both the Heterotrait–Monotrait ratio of correlations (HTMT), adopting the strict threshold of 0.85 [91], and the Fornell–Larcker criterion. Overall model fit was evaluated using the Standardized Root Mean Square Residual (SRMR), with values below 0.10 considered acceptable [89] and values below 0.08 indicative of good fit [91]. The structural model was evaluated by examining the magnitude and statistical significance of path coefficients (β), the coefficient of determination (*R*^2^) for endogenous constructs, and effect sizes (*f*^2^) to determine the practical impact of predictors. Specific indirect effects and serial mediation pathways were assessed to evaluate mediating mechanisms, with bootstrapped confidence intervals used to test the significance of indirect effects. To account for potential confounding effects, sociodemographic variables were included as control variables. To exclude serious common-method bias (CMB), Harman’s single-factor test [92] was conducted. Statistical analysis was performed with R (4.5.3).

## 3. Results

### 3.1. Measurement Model

The measurement model (Table 1) was evaluated by examining indicator reliability, internal consistency reliability, convergent validity, and discriminant validity. Factor loadings for most indicators met or exceeded the recommended threshold of 0.70. Specifically, faculty support items reported strong loadings (0.69–0.87), while self-compassion items ranged from 0.48 to 0.84. Career self-efficacy indicators showed loadings between 0.60 and 0.73, career planning items ranged from 0.38 to 0.87, and perceived employability indicators ranged from 0.67 to 0.78.

In the present study, all items of each construct were retained in accordance with their theoretical structure, despite some outer loadings falling below the conventional thresholds. Specifically, for self-compassion, the 10 scoring items of the SC-CEAS were retained in accordance with the original structure of the scale and the Italian validation by Nerini et al. [86], which considered items 3 and 7 of the Engagement subscale and item 3 of the Action subscale as non-scoring filler items. In an initial PLS-SEM estimation including all 13 SC items, SC.3 (λ = 0.06, *p* = 0.61), SC.7 (λ = 0.06, *p* = 0.60), and SC.11 (λ = 0.27, *p* = 0.01) showed near-zero or substantially subthreshold loadings, empirically confirming their non-scoring filler status. Therefore, their exclusion does not compromise construct validity, as these items are explicitly not intended to be scored and serve only as attentional probes during the administration of the scale [62,86]. Although SC.4 showed an outer loading of 0.48, its removal would not have been theoretically supported. The item specifically captures the capacity to tolerate distressing feelings—a facet of the Engagement dimension that Gilbert et al. [62] conceptualize as theoretically irreducible to attentional sensitivity and empathic insight, and therefore non-redundant within the subscale structure. Excluding it would result in underrepresentation of the Engagement dimension in the content, violating the theoretical boundaries of the construct [93]. Consistent with this reasoning, the Turkish validation of the CEAS [94] retained items 4 and 8 despite suboptimal loadings, arguing that they assess theoretically irreplaceable aspects of self-compassion—namely, the tolerance of distressed emotions. Furthermore, the Italian CFA by Nerini et al. [86] confirmed that Engagement items systematically display lower item-total correlations than Action items (range 0.41–0.59 vs. 0.52–0.60), a pattern attributable to the broader, multifaceted nature of the Engagement dimension rather than to item inadequacy. Notably, in that validation, item 4—capturing distress tolerance—yielded a corrected item-total correlation of 0.37, and item 8 yielded 0.55, both within the range documented for Engagement items and consistent with the present findings.

In our study, AVE of the self-compassion construct (0.50) met the minimum threshold proposed by Fornell and Larcker [95] (Table A1), and composite reliability (CR = 0.90) and rho_A (0.90) both substantially exceeded the recommended 0.70 threshold, confirming construct reliability.

Regarding career self-efficacy, all seven items of the scale were retained despite outer loadings ranging from 0.60 to 0.73 and an AVE of 0.46. Although this value falls below the conventional 0.50 threshold, Fornell and Larcker [95] suggested that if AVE is less than 0.50 but composite reliability exceeds 0.60, the convergent validity of the construct should be considered adequate. In the present study, composite reliability (CR = 0.85) and rho_A (0.82) exceeded this criterion. This approach has been applied in prior PLS-SEM research—for instance, Lam [96] reported and defended AVE values below 0.50 and CR values above 0.60. Furthermore, Hair et al. [97] indicate that a Cronbach’s alpha ≥ 0.60 constitutes a minimum acceptable reliability threshold; the present scale (α = 0.81) substantially exceeds this criterion. In addition to this statistical justification, career self-efficacy, as conceptualized by Lent et al. [22], is an inherently multidimensional construct consisting of distinct but related task-specific confidence beliefs—goal setting, occupational information gathering, planning, problem-solving, and self-appraisal. These behavioral domains are theoretically correlated but not interchangeable; their moderate loadings on a single latent factor are therefore expected and documented across validation studies of career self-efficacy instruments [98]. Removing lower-loading items would reduce content coverage without theoretical support.

Similarly, for career planning, all six items were retained, including PLAN.1 (λ = 0.40) and PLAN.5 (λ = 0.38), despite their substantially lower loadings relative to the core items (PLAN.2–PLAN.4, range 0.83–0.87). As with career self-efficacy, the Fornell and Larcker [95] exception applies: the AVE of 0.46, while below the conventional threshold, is accompanied by a composite reliability (CR = 0.82) well above 0.60, confirming adequate convergent validity. Moreover, the construct is a multiphase process construct comprising both distal cognitive components—awareness of the need to plan and general orientation toward the future—and proximal behavioral components such as active information search and concrete decision-making [99]. The two lower-loading items capture the attitudinal and distal phases of planning, which, while less closely related to the behavioral core, constitute a theoretically necessary component of the full construct. Their removal would bias the latent variable toward the purely behavioral dimension, leading to construct-level misspecification [92].

Internal consistency was deemed adequate across all constructs (α ≥ 0.79; Rho_A ≥ 0.80; CR ≥ 0.82). Cronbach’s alpha exceeded 0.70 for all constructs, including faculty support, self-compassion, career self-efficacy, career planning, and perceived employability. Convergent validity was evaluated through Average Variance Extracted (AVE). Faculty support (AVE = 0.65), perceived employability (AVE = 0.54), and self-compassion (AVE = 0.50) exceeded or met the 0.50 threshold. Career self-efficacy (AVE = 0.46) and career planning (AVE = 0.46) fell slightly below the conventional 0.50 benchmark but were retained given that AVE values between 0.36 and 0.50 are considered acceptable when composite reliability exceeds 0.70 [95], a condition satisfied in this study (CR ≥ 0.82 for all constructs). Discriminant validity was assessed via the HTMT criterion [91], which has been shown to be more robust than the Fornell–Larcker criterion in PLS-SEM contexts and does not rely on AVE. All HTMT values remained below the strict threshold of 0.85, with the highest value observed for the relationship between career self-efficacy and self-compassion (HTMT = 0.64). Additionally, the square root of each construct’s AVE exceeded its correlations with other constructs, further confirming discriminant validity according to the Fornell–Larcker criterion. Collinearity statistics were examined, and all predictors had VIF values below 3.0, indicating no significant multicollinearity (Table A2). Finally, the results of the Harman single-factor test revealed that the single factor accounted for 25.90% of the total variance (see the Appendix A). Because this value is well below the recommended threshold of 50%, CMB does not appear to be a pervasive issue in this study. These results, together with the structural coefficients, support the interpretability of the measurement model.

### 3.2. Structural Model

Since a satisfactory measurement model was ascertained, hypotheses were formally evaluated with the structural model of PLS-SEM (Table 2). The saturated model yielded an SRMR of 0.08, whereas the estimated model yielded an SRMR of 0.09. Path coefficients were assessed for their magnitude, statistical significance, and 95% confidence intervals through bootstrapping with 10,000 subsamples.

Prior to testing the hypothesized paths, the effects of sociodemographic control variables on perceived employability were examined showing that age (β = 0.17, *t* = 2.77, *p* = 0.006, 95% CI [0.04, 0.28]) and socioeconomic status (β = 0.16, *t* = 2.45, *p* = 0.014, 95% CI [0.03, 0.28]) significantly predicted perceived employability.

Findings confirmed the majority of the hypothesized relationships (Table 2). Specifically, perceived support was positively associated with self-compassion (*p* < 0.001) and career self-efficacy (*p* = 0.02) but was not significantly associated with career planning (*p* = 0.47) or perceived employability (*p* = 0.08). Self-compassion demonstrated a strong positive association with career self-efficacy (*p* < 0.001), whereas it did not show significant direct effects on perceived employability (*p* = 0.94) or career planning (*p* = 0.44).

Career self-efficacy was positively associated with both perceived employability (*p* < 0.001) and career planning (*p* < 0.001), while career planning significantly predicted perceived employability (*p* < 0.001). Regarding the variance explained, the model accounted for 48.7% of the variance in perceived employability, 33.8% in career self-efficacy, 20.0% in career planning, and 13.5% in self-compassion.

Mediation analyses were conducted to verify the hypothesized mediating effects. Results revealed significant indirect effects through both single and serial mediation pathways. Specifically, the indirect effect of self-compassion on employability through career self-efficacy was significant (*p* < 0.001), as was the serial mediation through career self-efficacy and career planning (*p* = 0.01). Faculty support showed significant indirect effects on employability through multiple pathways: via self-compassion and career self-efficacy (*p* = 0.01), via career self-efficacy directly (*p* = 0.04), and through the complete serial mediation chain involving self-compassion, career self-efficacy, and career planning (*p* = 0.04). The total effect of faculty support on employability was statistically significant (*p* < 0.001). Career self-efficacy also demonstrated a significant indirect effect on employability through career planning (*p* = 0.01), indicating partial mediation.

## 4. Discussion

The findings of the present study provide empirical support for a multidimensional view of perceived employability among university students. Specifically, the findings suggest that, in the current volatile labor market, employability emerges not from isolated factors but from a complex resource caravan, as suggested by the COR theory [63].

Regarding our findings, H1d was not supported, as faculty support did not show a direct association with perceived employability. This result contrasts with prior studies [20] and the SCCT [22], which identify social support as a primary source for career development. However, it is worth noting that previous research has primarily focused on family support or support from significant others, whereas only a limited number of studies have highlighted the specific role of instructors in promoting students’ career self-efficacy [77,100]. In this regard, Di Fabio and Kenny [101] showed that contextual factors, such as academic instructional support and access to university resources, strengthen students’ career self-efficacy and promote the exploration of a wide range of career opportunities. Our result could be interpreted considering the specific manifestation of support considered: instructors’ autonomy-supportive behaviors [76]. Such support is aimed at fostering personal agency and self-directed engagement rather than providing direct instrumental aid or ready-made career solutions. Consequently, autonomy-supportive instructors may not directly affect students’ perceived employability. This specific environmental resource, by creating a climate that validates students’ autonomy, promotes the accumulation of proximal, internalized psychological resources—specifically, career self-efficacy (H1b) and self-compassion. These internalized cognitive and emotional assets subsequently mobilize proactive behavioral strategies, such as career planning, which in turn enhance perceived employability. This sequential activation aligns with both the SCCT [22] and the perspective of Vanhercke et al. [13], who argue that self-perceived employability emerges from the dynamic interaction between individual characteristics and contextual factors and should therefore be conceptualized as a holistic construct.

Regarding the emotional dimension, this mechanism is in line with H1a and finds theoretical support in Gilbert et al.’s [62] approach to compassion, which suggests that the experience of receiving validation and safe relational support from external figures—such as autonomy-supportive instructors—is progressively internalized by students, transforming into self-directed kindness.

Regarding H2b and H2c, the expected direct paths from self-compassion to career planning and perceived employability were not confirmed. While some previous research found direct associations between self-compassion and positive career evaluations—such as Shin [18], who found that self-compassion was associated with lower employment anxiety—this absence of direct effects can be explained by considering the specific challenges of the Italian labor market. As it is a context characterized by high uncertainty and a pervasive skills mismatch, an emotional resource like self-compassion, though essential for reducing distress, cannot, on its own, automatically generate concrete behavioral strategies or a sense of labor market readiness. Within the SCCT and COR frameworks, self-compassion may serve as a foundational factor in effective emotional regulation, helping prevent energy depletion. It provides the necessary psychological safety to foster domain-specific confidence, but it must be translated into a cognitive asset to drive action.

In support of this thesis, the findings support H2a, suggesting that self-compassion may be a factor associated with increased career self-efficacy. This path is consistent with the literature documenting its potential to promote perceived self-efficacy among students [18,34,59,102]. This can be explained by the role of self-compassion in enabling a more balanced evaluation of personal limitations, reducing self-criticism and over-identification with negative emotions, and thereby sustaining a more positive perception of one’s capabilities [57]. Hence, when students adopt a self-compassionate attitude, they are less likely to be paralyzed by the fear of academic or professional failure. In other words, self-compassion can preserve the cognitive resources necessary for students to objectively appraise their skills by reducing harsh self-criticism and employment-related anxiety [18]. Such an emotional regulation mechanism, in turn, can enhance students’ confidence in their ability to successfully manage career transitions, or career self-efficacy, as documented by Liao et al. [102].

Regarding the latter construct, our results confirm the crucial role of career self-efficacy in the career development process, as it emerged positively associated with both career planning (H3a) and perceived employability (H3b). In this regard, several studies have shown that self-efficacy is intrinsically associated with perceived employability [28,52,103,104]. This link is well described by SCCT, which views efficacy beliefs as a motivational engine [37]. Therefore, when students are confident in their ability to successfully manage career-related tasks, they are more likely to translate this confidence into goal-directed, proactive behaviors, such as formulating clear career strategies and setting specific objectives. These behaviors reflect career planning and are functional in increasing perceived employability, as highlighted in prior studies and our findings (H4). Therefore, it can be stated that students who actively organize their career choices, gather relevant occupational information, and develop strategies to meet labor market demands may accumulate crucial behavioral resources and be perceived as having greater labor market readiness.

Furthermore, the hypothesized serial mediation (H5) was confirmed. Specifically, the findings suggest that the autonomy-oriented support considered, rather than directly affecting perceived employability, acts as a factor that triggers the accumulation and strengthening of personal resources, consistent with the COR theory [49]. From this perspective, faculty support may activate resource-gain cycles that sustain adaptive processes relevant to university students’ career development by promoting an academic climate that encourages the internalization of self-compassion [62]. The latter, by buffering against distress and self-criticism [57], preserves the psychological energy required to increase efficacy beliefs [102]. Subsequently, this heightened career self-efficacy drives students to engage in proactive career planning, which, in turn, can enhance their perceived employability, making them feel better equipped to navigate the uncertainty of the labor market [13,34].

Some limitations should nevertheless be acknowledged. First, the cross-sectional design of the study does not allow any inference about the direction of the observed associations. Consequently, an interpretative limit of the mediation analysis must be recognized: the serial pathways that emerged reflect only the theoretical framework adopted rather than an empirically established temporal chronology. Second, the small, non-representative sample limits the generalizability of the findings. Third, although the Harman single-factor test suggested that this bias was not pervasive, potential common-method bias should be considered. Fourth, the AVE values for two constructs fell slightly below the recommended threshold, resulting in a psychometric boundary that should be acknowledged. Additionally, for instruments lacking an established Italian validation, the absence of a formal back-translation procedure constitutes a potential methodological limitation. Given these considerations, future studies should employ longitudinal designs to examine more rigorously the temporal ordering of the variables involved. It would also be important to include students from a wider range of Italian universities and disciplinary fields to test the patterns of these associations across different academic contexts. Further research may also explore additional contextual and personal resources that could be relevant to the development of employability during the transition from university to work, such as peer networks and students’ sense of community [68,69]. Finally, it might be useful to integrate self-report measures with objective or behavioral indicators (e.g., academic records, observer-rated faculty support).

## 5. Conclusions

The study suggests that students’ perceived employability should be considered the result of a configuration of emotional, cognitive, and behavioral resources, rather than the isolated contribution of single factors. Specifically, contextual support and self-regulation skills emerge as essential in shaping this belief. In this vein, while academic organizations cannot directly grant labor market readiness, they play a crucial role in fostering the emotional regulation and confidence necessary for students to proactively design their professional futures.

From an applied and preventive standpoint, these insights call for a broader educational approach to career preparation. Rather than focusing exclusively on disciplinary knowledge or isolated counseling services, higher education institutions should implement systemic interventions. Specifically, the latter should include adopting autonomy-supportive teaching practices in faculty development programs, embedding self-compassion modules into the curriculum, and integrating career planning activities earlier in the curriculum rather than only close to graduation. This approach could equip students with the skills needed to manage occupational uncertainty and, in turn, preserve their well-being.

## Figures and Tables

**Table 1:** Factor loadings, internal reliability, and AVE of the measurement model.

Item	Mean	SD	Range	SUPP	SC	CEFF	PLAN	EMPL
**SUPP.1**	4.58	1.36	1–7	0.82				
**SUPP.2**	4.52	1.22	1–7	0.87				
**SUPP.3**	4.28	1.48	1–7	0.85				
**SUPP.4**	5.10	1.34	1–7	0.69				
**SUPP.5**	4.76	1.38	1–7	0.77				
**SUPP.6**	4.98	1.25	1–7	0.82				
**SC.1**	7.23	1.50	1–10		0.74			
**SC.2**	6.86	1.57	1–10		0.84			
**SC.4**	7.26	1.76	1–10		0.48			
**SC.5**	6.68	1.68	1–10		0.82			
**SC.6**	7.05	1.64	1–10		0.78			
**SC.8**	7.67	1.86	1–10		0.59			
**SC.9**	6.94	1.49	1–10		0.77			
**SC.10**	6.46	1.54	1–10		0.61			
**SC.12**	7.69	1.69	1–10		0.68			
**SC.13**	6.09	2.26	1–10		0.63			
**CEFF.1**	3.38	0.99	1–5			0.63		
**CEFF.2**	3.80	0.91	1–5			0.70		
**CEFF.3**	3.43	1.03	1–5			0.70		
**CEFF.4**	3.67	1.01	1–5			0.69		
**CEFF.5**	3.80	0.80	1–5			0.68		
**CEFF.6**	3.50	0.89	1–5			0.73		
**CEFF.7**	2.25	0.99	1–5			0.60		
**PLAN.1**	2.37	1.26	0–4				0.40	
**PLAN.2**	3.02	1.12	0–4				0.83	
**PLAN.3**	2.98	1.03	0–4				0.87	
**PLAN.4**	3.16	1.04	0–4				0.86	
**PLAN.5**	2.22	1.28	0–4				0.38	
**PLAN.6**	2.58	1.16	0–4				0.52	
**EMPL.1**	3.65	0.92	1–5					0.74
**EMPL.2**	2.58	1.14	1–5					0.67
**EMPL.3**	2.72	1.17	1–5					0.70
**EMPL.4**	3.36	1.04	1–5					0.78
**EMPL.5**	3.48	0.99	1–5					0.77
**Cronbach’s α**				0.89	0.88	0.81	0.79	0.79
**Rho_A**				0.90	0.90	0.81	0.89	0.80
**CR**				0.92	0.91	0.86	0.82	0.85
**AVE**				0.65	0.50	0.46	0.46	0.54

Notes: SUPP = Faculty support; SC = Self-compassion; CEFF = Career self-efficacy; PLAN = Career planning; EMPL = Perceived employability; α = Cronbach’s alpha; Rho_A = Dijkstra–Henseler’s rho; CR = composite reliability (Dillon–Goldstein’s rho); SD = Standard deviation; AVE = Average variance extracted.

**Table 2:** PLS-SEM results for hypothesis testing.

Path	Β	SE	CI
***Direct effects***			
SUPP → SC	0.37 **	0.07	[0.21, 0.49]
SUPP → CEFF	0.18 *	0.08	[0.02, 0.33]
SUPP → PLAN	0.07	0.09	[−0.12, 0.24]
SUPP → EMPL	0.12	0.07	[−0.02, 0.25]
SC → CEFF	0.49 **	0.06	[0.37, 0.59]
SC → PLAN	0.07	0.09	[−0.12, 0.25]
SC → EMPL	−0.01	0.08	[−0.16, 0.15]
CEFF → PLAN	0.37 **	0.08	[0.19, 0.51]
CEFF → EMPL	0.36 **	0.09	[0.19, 0.54]
PLAN → EMPL	0.30 **	0.07	[0.16, 0.43]
***Indirect effects***			
SUPP → SC → CEFF	0.18 **	0.05	[0.10, 0.27]
SUPP → SC → CEFF → EMPL	0.07 **	0.02	[0.03, 0.12]
SUPP → CEFF → EMPL	0.07 *	0.03	[0.02, 0.14]
SUPP → CEFF → PLAN	0.07 *	0.03	[0.01, 0.14]
SUPP → CEFF → PLAN → EMPL	0.02	0.01	[0.00, 0.05]
SUPP → SC → CEFF → PLAN	0.07 **	0.03	[0.03, 0.12]
SUPP → SC → CEFF → PLAN → EMPL	0.02 *	0.01	[0.01, 0.04]
SC → CEFF → EMPL	0.18 **	0.05	[0.09, 0.29]
SC → CEFF → PLAN	0.18 **	0.05	[0.09, 0.27]
SC → CEFF → PLAN → EMPL	0.05 **	0.02	[0.02, 0.10]
CEFF → PLAN → EMPL	0.11 **	0.04	[0.05, 0.20]
***Total effect***			
SUPP → EMPL	0.32 **	0.08	[0.14, 0.48]

Notes: SUPP = Faculty support; SC = Self-compassion; CEFF = Career self-efficacy; PLAN = Career planning; EMPL = Perceived employability; SE = standard error; CI = confidence interval. * *p* < 0.05, ** *p* < 0.01.

## Data Availability

The dataset that supports the findings of this study is available from the corresponding author upon reasonable request.

## Appendix A

**Table A1:** Discriminant validity with the Fornell–Larcker criterion.

Construct	SUPP	SC	CEFF	PLAN	EMPL
**SUPP**	**0.80**				
**SC**	0.37	**0.70**			
**CEFF**	0.36	0.56	**0.68**		
**PLAN**	0.23	0.30	0.44	**0.68**	
**EMPL**	0.36	0.37	0.57	0.52	**0.73**

Notes: SUPP = Faculty support; SC = Self-compassion; CEFF = Career self-efficacy; PLAN = Career planning; EMPL = Perceived employability. Values in bold on the diagonal represent the square root of the Average Variance Extracted (AVE). Discriminant validity is established as each diagonal value is higher than all other correlations in the corresponding row and column.

**Table A2:** Structural model—Multicollinearity check (Variance Inflation Factors—VIFs).

Predictor	CEFF	PLAN	EMPL
**SUPP**	1.16	1.21	1.31
**SC**	1.16	1.52	1.58
**CEFF**	—	1.51	2.13
**PLAN**	—	—	1.33

Notes: SUPP = Faculty support; SC = Self-compassion; CEFF = Career self-efficacy; PLAN = Career planning; EMPL = Perceived employability.
